# Supplementary material for: Are carbon emissions trading and green financial instruments synergistic? -Comprehensive quantitative research based on content analysis
Source: PLoS One. 2024 Mar 7;19(3):e0298601. doi: 10.1371/journal.pone.0298601 (PMC10919601; doi:10.1371/journal.pone.0298601)
Supplement: S1 Data — (ZIP) [file pone.0298601.s001.zip › Data/地级市Z_F工作报告.zip!/╒π╜¡/╔▄╨╦╩╨/└┤╫╘╣1⁄2╓┌║┼í╛┬φ┐╦╩2╛▌═°í┐_2018.docx]

各位代表：

　　现在，我代表绍兴市人民政府向大会作工作报告，请予审议，并请市政协委员和其他列席人员提出意见。

2017年工作回顾

　　2017年是深入实施“十三五”规划的重要一年，也是本届政府的开局之年。在中共绍兴市委的坚强领导下，市政府认真学习贯彻党的十九大精神，坚持以“八八战略”为总纲，全面落实省、市党代会决策部署，以打好“八大战役”为抓手，稳中求进、砥砺前行，较好地完成了市八届人大一次会议确定的目标任务。全市实现生产总值5108亿元，增长7.1%；一般公共预算收入431.4亿元，增长11.9%；城镇和农村常住居民人均可支配收入54445元、30331元，分别增长8.2%和9.3%。

　　一年来，我们立足换届新起点，着力在一些事关全市发展大局的重点领域和方面下功夫。一是重抓开局谋划新篇，积极抢抓全省大湾区大花园大通道大都市区建设机遇，科学编制绍兴行动方案，高起点推进绍兴科创大走廊、文创大走廊和环杭州湾产业带规划建设，全力推动重大战略部署在绍兴落地生根。二是重抓经济平稳回升，围绕振兴实体经济，出台加快转型升级提升绍兴产业品质等政策意见，统筹做好政府引导、企业帮扶和风险防控工作，着力优化投资结构、稳定外贸出口、促进消费升级，推动经济实现较快恢复性增长。三是重抓新旧动能转换，扎实推进传统产业和开发区（工业园区）改造提升、上市公司引领产业发展示范区建设省级试点，制定出台培育发展新兴产业三年行动计划，重点传统产业产销和利税实现两位数增长，引进中芯国际集成电路、电咖新能源汽车、安吉尔环境、德升动力电池三元材料等一批投资50亿元以上重大新兴产业项目。四是重抓改革攻坚破难，全面推行“一窗受理、集成服务”，“最多跑一次”事项实现率居全省前列，柯桥投资项目审批集成式改革列入省级试点，诸暨“一证通办一生事”经验全省推广；深入推进市与区事权调整改革，将753项权力事项划归越城区行使，规划、国土、环保实现垂直化管理。五是重抓城市品质提升，对标杭州G20峰会整治标准，高质量实施城区景观提升工程，成功举办首届绍兴国际马拉松赛和2017中国·绍兴皮划艇马拉松世界杯赛，成功蝉联全国文明城市，绍兴美誉度、影响力进一步提升。六是重抓民生办好实事，坚持问政于民、问需于民、问计于民，持续加大民生投入，十方面民生实事全面完成，群众获得感和满意度不断提升。

　　经过一年来全市上下的共同努力，各方面工作取得积极成效。

　　（一）产业层次稳步提升。工业结构调整优化，39个项目列入省“百项万亿”重大制造业发展计划，成为首批国家通航产业示范区、首批省军民融合创新示范区，全市规上工业增加值增长7.9%，其中规上新兴产业增加值增长9.4%、占比提高到33.3%。新增工业机器人1598台、上云企业超过1万家。新增上市公司9家、新三板挂牌企业29家，培育认定“隐形冠军”企业210家，实现“小升规”企业693家。实体经济发展环境不断改善，累计为企业减负125.7亿元、转贷963亿元，整治提升印染化工企业372家、工业园区452家，淘汰354家企业落后产能，处置僵尸企业47家、不良贷款253.1亿元。建筑产业稳健发展，新开工装配式建筑309.2万平方米，施工总承包特级企业增加到19家，建筑业总产值增长7.4%。服务业保持较快发展，文化旅游、创意设计、健康养生等现代服务业逐步壮大，服务业增加值增长8%，社会消费品零售总额增长10.9%。全域旅游示范区创建积极推进，新昌列入全国创建名录，东方山水二期、穿岩十九峰二期等项目启动建设，旅游总收入增长15.4%。

　　（二）发展动力不断增强。坚定实施创新驱动发展战略，新昌全面创新改革试验区建设扎实推进，浙江“****”绍兴产业园加快建设，绍兴科技大市场开业运营，规模10亿元的科技创新基金设立运作，建成省级众创空间14家、科技孵化器28家，被列为全省唯一市级全面创新改革联系点。新增国家高新技术企业220家，省级科技型中小企业数量增长40.1%，规上工业新产品产值增长26%，新产品产值率居全省第二。联动推进质量、标准、品牌建设，成功创建全国质量强市示范城市。制定出台“打赢人才招引战”政策举措，新增国家和省级院士工作站4家、“国千”“省千”及海外高层次人才225名。“放管服”改革纵深推进，“多证合一、一照一码、证照联办”商事制度改革不断深化，率先在全省实现“中介超市”全市域互联互通，新增各类市场主体8.9万家、增长26.5%。国企改革有序推进，交投集团、文旅集团等完成整合重组。制定实施深度参与“一带一路”建设行动方案，“单一窗口”等便利化举措稳步推进，首批“品质浙货”出口领军企业占全省比重达到20%，实现外贸出口1852亿元、增长9.8%。积极扩大有效投资，完成固定资产投资3115.7亿元、增长11.1%，引进10亿元以上先进制造业项目27个，实到市外资金519.3亿元，实际利用外资12.4亿美元、增长55.2%。对口支援、对口合作和山海协作等工作扎实开展。

　　（三）城市建设有力推进。强化规划引领，科学编制新一轮城市总体规划，扎实推进镜湖核心区城市设计，海绵城市等专项规划编制完成。诸暨“北承南接”战略全面实施，成功创建县级全国文明城市。嵊新区域协同发展不断深化，通道经济发展规划启动编制。基础设施建设协调推进，杭绍台铁路、金甬铁路、杭绍台高速等项目有序实施，绍诸高速诸暨延伸线建成投用，常台高速新昌南互通项目开工建设；绍兴平原和两江流域“双十”防洪排涝工程积极推进，新昌钦寸水库、柯桥平原排涝快速通道、上虞世纪新丘治江围涂等工程全面建成。中心城市建设步伐加快，三区融合年度任务全面落实，非古城功能有序疏解，12个镇完成撤镇建街，轨道交通1号线开工建设，杭绍城际铁路、群贤路东延、绍三线北延、32省道改造等项目建设积极推进，风情旅游新干线即将开行，104国道柯桥段高架主线、上虞客运中心建成投用。

　　（四）农业农村持续发展。品质农业建设稳步推进，完成“双十大”投资8亿元，创建全国农村“双创”示范基地5家，粮食生产保持稳定，农产品检测合格率达到99.3%。村庄布点、建设等专项规划编制完成。基本建成新“十大美丽乡村景观带”，建成美丽乡村建设示范镇31个、特色精品村61个、A级以上景区村234个，乡村休闲旅游收入增长18%。农村基础设施持续完善，提升农村等级公路228公里，建设美丽交通走廊476公里，在全省率先实施城乡公交通达标准并轨，柯桥成为全国首批“四好农村路”示范区，嵊州、新昌完成城乡客运一体化改革，实现公共区域视频监控“村村通”。涉农改革纵深推进，农地确权、农房发证进度全省领先。完成农民培训8.3万人次，集体经济年收入10万元以下薄弱村全面消除。

　　（五）生态环境不断改善。扎实开展国家生态文明建设示范市创建，新昌成为全国首批生态文明建设示范县。中央环保督察反馈问题整改工作有力推进，生态环境损害赔偿制度、监测监察执法垂直管理等改革试点深入实施，生态红线调整优化，污染源普查全面启动。“治污剿劣”成效明显，新建城镇污水配套管网431.4公里，实现建制村生活污水治理全覆盖，全面剿灭2752个劣Ⅴ类小微水体，市级考核断面Ⅲ类以上水质比例达到96.9%。“河长制”管理不断深化，“湖长制”管理率先全覆盖。大气污染防治三年行动计划深入实施，PM2.5浓度下降8.9%，空气优良天数比例达到83%，重污染天气基本消灭。土壤污染防治方案制定实施。改造旧住宅区、旧厂区、城中村8557万平方米，拆违2375万平方米，城乡D级危旧房治理改造全面完成，小城镇环境综合整治工作居全省前列。垃圾源头减量与分类处置水平不断提升，市区城区、农村生活垃圾分类覆盖率分别达到83.2%和71%，循环生态产业园一期再生资源发电厂项目基本建成。完成国土绿化“十大”工程投资19.2亿元。

　　（六）社会事业协调发展。就业和社会保障水平不断提高，新增城镇就业12.5万人，城镇登记失业率2.25%，养老金提标稳步实施，最低月工资标准提高到1800元，实现低保标准全市城乡统一、生育保险制度全市统一、医保个人账户家庭共济和异地就医直接结算。学前教育向优质普惠发展，市区公办普通高中实现部分打通招生、重点特色学科面向全市招生，中小学教师“县管校聘”改革率先推开，越城、诸暨通过教育基本现代化县验收，绍兴一中新校区、新昌技师学院、浙江理工大学科艺学院投入使用。健康绍兴行动启动实施，嵊州、新昌创建成为国家卫生城市（县城），市县优质医疗资源下沉实现全覆盖，市立医院、市口腔医院、柯桥妇保院等建成投用，新增床位1650张。国家智慧居家养老标准化试点通过验收。建成保障性安居工程住房3.7万套。基层文化建设得到加强，新增农村文化礼堂125个，新建乡镇（街道）图书分馆15个、文化分馆54个，实现国有景区、公共文化场所分层分类免费开放。绍兴健儿勇夺全运会13枚金牌、创历史最好成绩。“基层治理四平台”建设全省领先。社会治安管理持续强化，刑事案件、电信网络诈骗案件立案数分别下降32.8%和34.4%。国家食品安全示范城市创建扎实推进，应急管理体系不断完善，生产安全事故起数、死亡人数分别下降38.4%和26.7%。国家安全、国防动员、双拥、人防等工作得到新加强，民族宗教、外事侨务、对台事务、档案、史志工作及妇女儿童、红十字、残疾人等事业取得新成绩。

过去一年，我们努力加强政府自身建设。认真学习贯彻党的十九大精神，牢固树立“四个意识”和“四个自信”，严格落实党建工作主体责任，扎实推进“两学一做”学习教育常态化制度化。修订完善政府工作规则，出台加强政府自身建设若干规定。自觉向市人大常委会报告工作、向市政协通报情况，主动接受各方监督。完善建议提案牵头领办和绩效跟踪机制，积极有效回应群众诉求。有序推动地方立法，积极实施行政复议体制改革。制定社会公众代表列席市政府会议制度，推进政务公开。强化预算执行管理与审计监督，探索实施公共产品集中采购、线上交易机制，全市“三公”经费支出下降29.7%。驰而不息推进作风建设，加强廉政风险防控与“庸懒散奢”整治，政府系统执行力、公信力不断提升。

　　各位代表，过去一年的成绩来之不易，是省委、省政府和市委正确领导，全市人民共同努力的结果。在此，我代表市人民政府，向全市人民，向给予政府工作大力支持的人大代表、政协委员、各民主党派、驻绍部队、武警官兵，向所有关心、支持、参与绍兴发展的同志们、朋友们，致以崇高的敬意和衷心的感谢！

　　我们也清醒地看到，经济社会发展和政府工作仍然存在不少问题，特别是发展不平衡不充分的问题还比较突出，主要表现在：经济发展质量不高，结构调整进度滞后于发展速度，创新能力与经济实力不够匹配；一些改革举措落地见效还不够快；中心城市功能布局不合理、基础设施不完善，集聚力不强；文化和旅游资源优势尚未充分转化为创新发展优势；生态环境仍比较脆弱；优质公共服务供给仍然不足，影响平安稳定的隐患还不少；一些党员干部能力水平和作风状况不适应新时代发展要求。对这些问题，我们将高度重视，采取切实有效的举措认真加以解决。

2018年主要工作

　　2018年是贯彻党的十九大精神的开局之年，是改革开放40周年，是决胜全面建成小康社会、实施“十三五”规划承上启下的关键一年，做好新一年政府工作意义重大。今年政府工作的总体要求是：高举习近平新时代中国特色社会主义思想伟大旗帜，全面贯彻党的十九大和十九届二中、三中全会精神，贯彻落实省第十四次党代会、省委十四届二次全会和市第八次党代会、市委八届二次全会部署，坚持和加强党的全面领导，坚持稳中求进工作总基调，坚持新发展理念，按照高质量发展的要求，坚定践行“八八战略”，牢固树立“四个强省”工作导向，坚决打好三大攻坚战，突出制造业强市、服务业兴市，突出质量结构效益，突出人文生态创新，全力打造东方水城，全面提升绍兴品质，促进经济持续健康发展和社会和谐稳定，为建设现代化强市奠定坚实基础。

　　全市经济社会发展主要预期目标是：生产总值增长7%左右，城乡居民收入增长、财政收入增长与经济增长基本同步，全员劳动生产率增长8%左右，研发经费支出与生产总值之比达到2.6%左右，节能减排降碳完成省下达目标。

　　实现上述目标，必须重点把握六个方面：一是聚焦改革开放，提升发展动力。认真总结改革开放40周年成功经验，以“最多跑一次”改革撬动各领域改革，以“一带一路”统领新一轮对外开放，持续优化发展环境，全力增创发展新优势。二是聚焦大湾区建设，提升产业品质。锚定大湾区高能级平台绍兴先进智造基地定位，强化产业联动、创新驱动、平台带动，推动产业有机更新、破旧立新，构建制造业、服务业双轮驱动现代产业体系，打造“万亩千亿”级产业新平台，努力建设新旧动能转换样板区。三是聚焦大花园建设，提升生态文明。按照“生产空间集约高效、生活空间宜居适度、生态空间山清水秀”要求，围绕国家生态文明建设示范市创建，全域推进“大景区”“大花园”建设，加快建设美丽乡村、美丽田园、美丽河湖、美丽城市、国家公园，持续提升生态文明建设水平。四是聚焦大通道建设，提升区位优势。主动融入义甬舟开放大通道，构建“两横、两纵、两对角”交通走廊，提高绍兴至周边核心空港、海港、陆港交通便捷度，深化与大都市区和主要功能平台之间的互联互通，加快打造全域开放、海陆联动的大通道枢纽城市。五是聚焦大都市区建设，提升城市能级。抢抓杭州、宁波一体化发展机遇，全力唱好“双城计”，加快中心城市建设，统筹诸暨、嵊新组群发展，推动新城古城联动发展，建立更加高效的全市域协同和城乡融合发展机制，全方位提升城市品质和竞争力。六是聚焦民生改善，提升保障水平。坚持以人民为中心的发展思想，紧扣社会主要矛盾变化，兜牢民生底线，不断优化公共服务和管理，加快形成共建共治共享的社会治理格局和富民惠民安民的政策保障体系，让改革发展成果更多更公平惠及全市人民。重点做好九方面工作：

　　（一）推动产业转型升级。坚持质量第一、效益优先，大力振兴实体经济，加快建设现代产业体系，全年规上工业增加值、服务业增加值分别增长7.5%和8%以上。

加快发展先进制造业。深入实施传统产业改造提升试点，重点传统产业产值增长8%以上。推动纺织业向时尚创意设计中心和创造中心转型，积极打造设计“梦工厂”和智造“云工厂”，加快印染企业集聚提升；推动化工业向生产清洁化、设备智能化、管理信息化发展，加快化工企业集聚入园；推动金属加工业向新材料、新产品、新技术、新应用领域拓展，着力建设国内有影响力的高端金属加工基地；推动黄酒业向创新品牌营销、拓展酒庄产业、融合文化旅游提升，加快建设黄酒小镇；推动珍珠业向珠宝设计、营销、展示方向发展，建设国家珠宝玉石质量监督检测中心。深化开发区（工业园区）改造提升试点，打好“四退四进”攻坚战，加快整合提升工业园区，全面推进“低小散”企业整治，淘汰300家企业落后产能，基本实现“僵尸企业”市场出清。加快上市公司引领产业发展示范区建设，深入实施“凤凰行动”计划，力争新上市8家、新三板挂牌12家、股改100家，兼并重组120亿元以上。围绕高端装备、新材料、现代医药、电子信息四大领域，深入实施培育发展新兴产业三年行动计划，深化军民融合创新示范区建设，新兴产业产值增长15%以上。积极引进工业互联网、大数据、人工智能等新技术新产业，发展数字经济。加快制造业技术改造，力争上云企业数量翻番，推广工业机器人1600台以上，新增“品字标”浙江制造标准30项以上。

大力发展现代服务业。加快实施服务业重大项目“百项千亿”工程，推进重点企业培育和集聚示范区建设。培育壮大文化旅游、健康养生、信息服务等重点行业，加快发展平台经济、分享经济、体验经济和创意经济。实施全域旅游示范区建设，谋划打造一批大景区和连线成片旅游线路，打好营销组合拳，推进新昌大佛寺、兰亭文化旅游区等创建国家5A级景区，积极创建鉴湖国家级旅游度假区，旅游总收入增长15%。深入开展“放心消费在绍兴”行动，培育发展重点商圈，加快打造特色商业街，推进批发零售业改造提升，开展电子商务创新发展省级试点，社会消费品零售总额增长10%左右。

　　做大做强现代住建业。推进国家装配式建筑示范城市建设，着力在建筑设计标准化、生产工厂化、施工装配化、装修一体化、管理信息化及新型绿色节能建材制造上求突破，加快建筑业转型升级。全年力争一级以上企业达到190家，新创“鲁班奖”或国优工程3项，装配式建筑占新建建筑比例达到20%以上。培育一批建设工程全过程咨询服务工作试点。完成公共建筑节能改造10万平方米。

　　（二）强化创新驱动发展。坚持把创新作为引领发展的第一动力，把人才作为支撑发展的第一资源，大力推广新昌全面创新改革试验工作经验，加快建设创新型城市。

　　培育壮大创新主体。深入实施高新技术企业和科技型中小微企业“双倍增”计划，力争新认定国家高新技术企业220家、省级科技型中小微企业1000家以上，新培育“隐形冠军”企业100家，高新技术产业投资增长10%以上。鼓励企业异地建设研发机构，推动大型工业企业研发机构建设、规上工业企业技术创新活动“两个全覆盖”。推广“一个产业引进建立一个研究院”模式，深化与大院名校科技合作，研发人员数达到3.9万人年，高新技术产业增加值增长8%、规上新产品产值增长10%。

　　推动创业创新平台建设。加快建设绍兴科创大走廊，扎实推进各类公共创新服务平台建设。积极打造纺织面料、领带、印染、化工、医药、金属加工、高端装备等产业创新服务综合体，助推产业优化升级。全面实施众创空间三年行动计划，鼓励社会力量建设创客空间、创新工场等新型众创空间，新增省级以上众创空间19家。深化国家知识产权示范城市创建，更好发挥科技大市场作用，加快推进科研成果产业化。

实施人才引育工程。围绕《高水平建设人才强市三年行动计划》，全面实施“十百千万”重大引才工程和“三个一批”人才培育工程，新建海内外引才工作站20家、院士专家工作站10家、海外创新孵化基地4家，新引进落户“国千”“省千”人才50名，培训优秀企业家100名，新增高技能人才3万名，新就业大学生5万名。深化实施人才新政，加强“一站式”人才综合服务平台建设，成立高层次人才服务联盟，建成海智汇·绍兴国际人才创业创新服务中心，积极打造人才生态最优市。

　　（三）推进全面深化改革。坚定不移高举改革大旗，不断增强改革的系统性、整体性、协同性，努力增创体制机制新优势。

　　纵深推进“最多跑一次”改革。围绕“一窗办、全城办、一证办、移动办”目标，深化“一窗受理、集成服务”改革。加快投资项目审批集成式和“多合一”改革，推动实现企业投资项目审批竣工验收前“最多跑一次”和“符合条件项目50天”“一般项目最多100天”。推进400个以上重点事项实现市区“同城通办”、90个以上民生事项实现“一证通办”。推行政务服务“移动办”，实现更多事项网上办理。

　　深化国资国企改革。制定市属国企三年发展规划，加快国有经济布局优化、结构调整、整合重组，抓好漓铁、平铜、华越等市属国企改革转型。积极发展混合所有制经济，提高国有资产证券化率。优化国资监管模式，推进经营性国资集中统一监管，实现市属国企外派监事会建设全覆盖。加强国企业绩清单化考核，落实任期制契约化管理，规范企业收入分配秩序，增强国有经济发展活力。

　　统筹抓好其他领域改革。完善城市管理体制机制，深化三区公共服务领域一体化改革。加强投融资体制改革，规范推广政府和社会资本合作（PPP）模式，大力激发民间投资活力，支持诸暨创业投资改革试验区建设。加快要素市场化配置改革，实施行业“亩产效益”领跑者计划，推行企业对标竞价的“标准地”制度，推进全市域区域能评、环评改革。

　　（四）提高开放发展水平。坚持引进来与走出去相结合，加强国际和区域开放合作，推动形成全面开放新格局。

　　积极拓展对外贸易。实施外贸优进优出战略，积极融入浙江自贸区建设，引导外贸出口向价值链中高端攀升，支持企业扩大先进技术设备和关键零部件进口，力促进出口稳中向好。开展加工贸易监管改革等试点，加快培育省级服务贸易发展基地，推进跨境电商产业集群建设。突出“一带一路”沿线国家和地区，加大市场拓展力度。实施千企贸易成长计划，培育文化服务贸易重点企业50家、年出口额超千万元服务外包企业20家以上。

　　持续发力精准招商。以实施“市县长项目工程”为主抓手，重点围绕先进制造业、现代服务业，谋划引进10亿元以上产业项目30个以上、50亿元以上产业项目8个以上，项目工程落地率达到50%以上，力争实际利用外资增长5%、实到市外资金增长10%，带动民间投资增长10%以上。

　　深化内外交流合作。实施本土民营跨国公司培育三年行动，鼓励纺织、化工等优势产业开展国际产能合作，推进中丹产业园、浙商跨国并购项目回归产业园等平台建设，支持企业参与“一带一路”基础工程建设。主动对接融入杭州、宁波两大都市区建设，全面深化产业、交通、文化、旅游、民生等各领域区域合作，努力实现左右逢源、同城发展。办好绍兴发展大会、第二届国际友城大会、中国集成电路发展高峰论坛和2018绍兴国际马拉松赛、皮划艇马拉松系列赛、女排俱乐部世锦赛，积极打造国际赛会目的地城市。继续做好对口支援、对口合作和山海协作等工作。

　　（五）提升城市能级品质。扎实推进绍兴大城市建设，着力提高中心城市能级，加快推动县域经济向城市经济转型，不断提升城市综合竞争力。

　　推进全市域协同发展。完成新一轮城市总体规划编制，建立城乡建设全域管控机制，推进“多规合一”。加强城市精细化管理，加快大数据平台建设，积极打造智慧城市。推进海绵城市试点建设。高质量培育小城市、建设特色小镇。完善城乡基础设施，开工建设杭绍台高速西线、绍兴南部综合交通枢纽、曹娥江“两闸一航道”等工程，加快推进金甬铁路、杭绍台铁路、杭绍台高速、527国道嵊州段新昌段、500千伏江滨输变电等项目，建成钱滨线、绍三线北延等项目，做好杭绍甬智慧高速、杭甬高速孙端互通、杭绍台铁路绍兴站综合枢纽等项目前期，交通投资增长10%以上。实施越东路、二环北路、329国道快速路改造，加快构建全市快速路网体系。

　　加快中心城市建设。高标准优化功能布局，加强重点区域城市设计，系统梳理中心城市空间形态、产业布局、交通组织和环境风貌，推动三区连接区域规划建设，加快越城柯桥板块、上虞板块融合发展。推进杭绍城际铁路、轨道交通1号线等项目建设，启动二环西路节点改造、凤林路东延、城南大道杨绍线拓宽改造等工程，建成群贤路东延二期、绸缎路和洋江路区间通道等项目。

　　加强镜湖核心区建设和古城保护利用。围绕打造全市首位度的城市核心和地标性新城区，编制实施镜湖核心区城市设计和控制性详细规划，完善开发建设模式，启动镜湖水环境综合整治，谋划推进新大剧院、博物馆、美术馆、规划展示馆、会展中心及高端教育、医疗、商业等公共设施建设。坚持世界文化遗产标准保护利用古城，加快疏解非古城功能，推进鲁迅故里、书圣故里、阳明故里等历史文化街区改造提升，加强古运河—鉴湖整治，实现古城景观提升基本全覆盖。

　　（六）实施乡村振兴战略。围绕“产业兴旺、生态宜居、乡风文明、治理有效、生活富裕”总要求，全面深化“五星达标、3A争创”工作，推动农业农村优先发展。

　　加快现代农业发展。深化农业“两区”建设，完善现代农业产业体系、生产体系、经营体系，促进农村一二三产业融合发展。加快农业产业集聚区和特色农业强镇建设，推进诸暨国家现代农业产业园、柯桥国家田园综合体试点，创建省级现代农业园区2个、美丽生态牧场21家，粮食总产量稳定在89万吨以上。积极培育农业区域品牌，鼓励农业龙头企业上市，新增省级以上品牌农产品10个，农业标准化率达到63%以上。实施农业种养殖类、农产品加工和农旅结合类项目20个，完成投资8亿元以上。加强农业科技创新，发展智慧农业，新增新型农机装备1万台套。

　　提高乡村建设水平。完成社区化改造村30个以上，推动实现60%的村五星达标、70个左右村达到3A级景区标准。加强农村人居环境整治，实现农村生活污水治理设施运维管理一体化。全面推进“四好农村路”建设，建成美丽经济交通走廊180公里，提升改造等外公路120公里，提前一年基本完成村村通公交三年计划。全面消除集体经济年收入不足15万元的薄弱村，村集体经济收入增长8%以上。推进农村土地“三权分置”改革，探索宅基地制度改革，完成柯桥全国农村集体产权制度改革试点，推广嵊州农村土地承包经营权抵押贷款试点经验。建立健全村规民约体系，完善自治、法治、德治相结合的治理体系。

　　促进农民增收致富。加快培育现代农民，全年培训新型职业农民和农村实用人才1.5万人。拓宽农民增收渠道，实施“闲置农房激活”计划，大力发展乡村旅游、农村电商、农家乐、民宿等新业态，鼓励大学生到农村创业。打好低收入百姓增收攻坚战，促进低收入农户收入增长10%以上。

　　（七）推进生态环境建设。牢固树立社会主义生态文明观，扎实开展美丽绍兴建设行动，加快推动生产生活生态融合发展。

　　打好污染防治攻坚战。深化“河湖长制”管理，创建“污水零直排区”，加快城镇污水处理设施提标改造，建成诸暨浣东再生水厂、嵊新污水处理厂二期等项目，确保市级考核断面水质全部达到或优于Ⅳ类水，功能区达标率达到92%以上。深入开展大气污染治理，完成38个印染、化工等行业VOCs治理示范工程，建成24个清新空气监测站点，建筑面积1.5万平方米以上工地全部安装在线监控系统，空气质量优良天数达到300天以上。加强重点行业企业用地土壤污染状况调查，完成现代农业园区土壤污染防治三年计划。深入抓好中央环保督察反馈问题的整改落实。全面推行三级政府生态环境报告制度。加大环保投入力度，生态环境和公共设施投资增长10%以上。

　　推动城市绿化彩化美化。市县联动实施城市景观提升工程，推进诸暨至越城道路、风情旅游新干线沿线环境提升。高标准深化小城镇环境综合整治，完成城中村和城市旧住宅区拆迁560万平方米以上，争创“基本无违建市”。全域建设国家森林城市，推进鉴湖国家湿地公园试点、会稽山国家森林公园创建，推进贺家池等重要生态系统保护修复。完成造林更新2万亩，新植珍贵树种165万株。

　　（八）保障和改善民生。牢牢把握人民群众对美好生活的向往，扎实办好民生实事、“关键小事”，不断提升人民群众获得感、幸福感和安全感。

　　优先发展教育事业。优化全市教育资源配置，实施学前教育第三轮行动计划，推进名校、名师、名专业建设，深化职业教育产教融合、校企合作，全面实现市区公办普通高中统一招生，确保教育基本现代化县全覆盖。大力发展高等教育，积极引进国内外知名院校，全力支持文理学院创建绍兴大学。整合提升绍兴艺校，规划建设绍兴体校、浙江建设职业技术学院上虞校区，开工建设绍兴技师学院、新聋哑学校。

　　加强就业和社会保障。完善支持大众创业促进就业政策，优化就业创业服务，新增城镇就业10万人以上，城镇调查失业率、登记失业率分别控制在5%和3%以内。实施全民参保计划，推进基本医保政策全市统一，深化医疗保险支付方式改革，完成机关事业单位养老保险制度改革。健全城乡统一的帮扶救助体系，大力发展红十字、慈善事业，积极推进市残疾人托养中心建设、市儿童福利院迁建。开展住房租赁市场培育省级试点，加快建立多主体供给、多渠道保障、租购并举的住房制度，促进房地产市场平稳健康发展。

　　加快建设健康绍兴。深化公立医院和基层卫生综合改革，推进上虞健康促进全国试点、柯桥医共体省级试点，谋划迁建市人民医院、市妇保院，确保通过国家卫生城市复审。鼓励扶持社会办医，推进医养结合。加强疾病预防控制，优化重点人群健康服务和生育全程服务。推进国家居家和社区养老服务改革试点，启动养老产业示范园区建设。广泛开展全民健身活动，建成2个大型游泳场馆，完成100个小康体育村升级，做好省运会备战参赛工作。

　　大力发展文化事业。弘扬社会主义核心价值观，深化全国文明城市建设。加快绍兴文创大走廊规划建设，推进大运河文化保护利用。加强“大禹文化”“浙东唐诗之路”“阳明心学”等传统文化资源研究开发，打造“书法进课堂”全国示范区，谋划建设宋六陵遗址公园，统筹办好第四届中国越剧艺术节、阳明心学高峰论坛等重大节会。振兴发展传统戏曲，推动文艺精品创作。完善文化产业政策，培育新型文化业态。推进文化惠民服务，深化“文化走亲”活动。加强古镇古村落保护利用。

　　推进社会治理创新。创新发展新时代“枫桥经验”，加强平安绍兴建设，深化“基层治理四平台”建设和网格化管理，开展“无信访积案市”创建，筹办好“枫桥经验”纪念活动。强化城市社区建设，全面开展“五星达标、和美家园”争创。打好金融风险防控攻坚战，加强不良资产处置和“两链”风险化解，坚决打击违法违规金融活动，进一步降低不良贷款率。加快社会治安防控体系建设，扎实开展扫黑除恶专项行动。加强消防、道路交通、食品药品等重点行业领域安全管理，完成现有地质灾害隐患点整治，提升防灾减灾和应急救援能力，坚决遏制重特大事故发生。深化“法润绍兴”行动，加强公共法律服务体系建设，基本建成公共信用信息平台。推进国家安全、国防动员、双拥、人防、民族宗教、外事侨务、对台事务等工作，支持工会、共青团、妇联、科协等人民团体建设。

　　今年，市政府将继续按照“群众提、代表定、政府办”的理念，认真办好十方面民生实事，着力解决人民群众普遍关心的突出问题。

　　（九）加强政府自身建设。坚持依法行政、为民务实、廉洁高效，突出“强谋划、强执行，提高行政质量、行政效率和政府公信力”要求，致力提高人民群众对政府工作的满意度。

　　建设学习型政府。扎实开展“不忘初心、牢记使命”主题教育，自觉用习近平新时代中国特色社会主义思想武装头脑，切实学懂弄通做实党的十九大精神。深入实施公务员知识更新三年轮训行动，加快提升政府工作人员知识水平和专业素养。大兴调查研究之风，坚持学中干、干中学，向实践学、向群众学，着力提高谋划大局、推动发展和解决实际问题的能力。

　　推进依法行政。深入实施法治政府建设纲要，依法全面履行政府职能。全面推进政务公开，自觉接受人大法律监督、政协民主监督，强化审计监督，主动接受社会监督、舆论监督。完善重大行政决策程序，加强新型智库建设，提高决策科学化、民主化、法治化水平。推动古城保护利用、古香榧群保护管理等方面立法，完善依法行政制度体系。深化公安、综合执法规范化建设，建立健全跨部门、跨行业联合执法机制，推行“双随机一公开”监管。

　　提升政府效能。推进政府运行体制改革和政府机构改革，深化简政放权和行政审批制度改革，加快转变政府职能。坚持一张蓝图绘到底、一任接着一任干、一以贯之抓落实，锲而不舍、久久为功，以钉钉子精神担当尽责。推行任务项目化、项目清单化，完善督查考核机制，增强政府执行力。加大清费减负、企业帮扶力度，公平高效落实社会事业和惠民政策，提高服务发展能力。提升财政资金绩效管理水平，加强政府性债务管控。推动政府治理数字化，加快建设政务大数据平台，推广无纸化办公。

　　加强廉政建设。严格落实中央八项规定及实施细则精神，认真履行各级政府和部门党组（党委）从严治党主体责任，严明党的纪律和规矩，持之以恒正风肃纪，防止“四风”问题回潮复燃。扎紧扎密制度笼子，进一步规范政府采购、工程招投标和政府投资行为。积极推进清廉绍兴建设，巩固发展反腐败斗争压倒性态势，坚决惩处各类腐败行为，努力营造政府系统风清气正的政治生态。

　　各位代表！新时代要有新气象，新征程要有新作为。让我们更加紧密地团结在以习近平同志为核心的党中央周围，高举习近平新时代中国特色社会主义思想伟大旗帜，在中共绍兴市委的坚强领导下，振奋精神、真抓实干，为高水平全面建成小康社会、加快建设现代化强市而努力奋斗！
